# Supplementary material for: The phased pan-genome of tetraploid European potato
Source: Nature. 2025 Apr 16;642(8067):389–97. doi: 10.1038/s41586-025-08843-0 (PMC12158759; doi:10.1038/s41586-025-08843-0)
Supplement: Supplementary file 5 — Supplementary Figs. 3–14, 20, 21, 23–27 and 34–44. [file 41586_2025_8843_MOESM5_ESM.zip › suppl_figure_34-44/suppl_figure_34_A_v3.pdf]

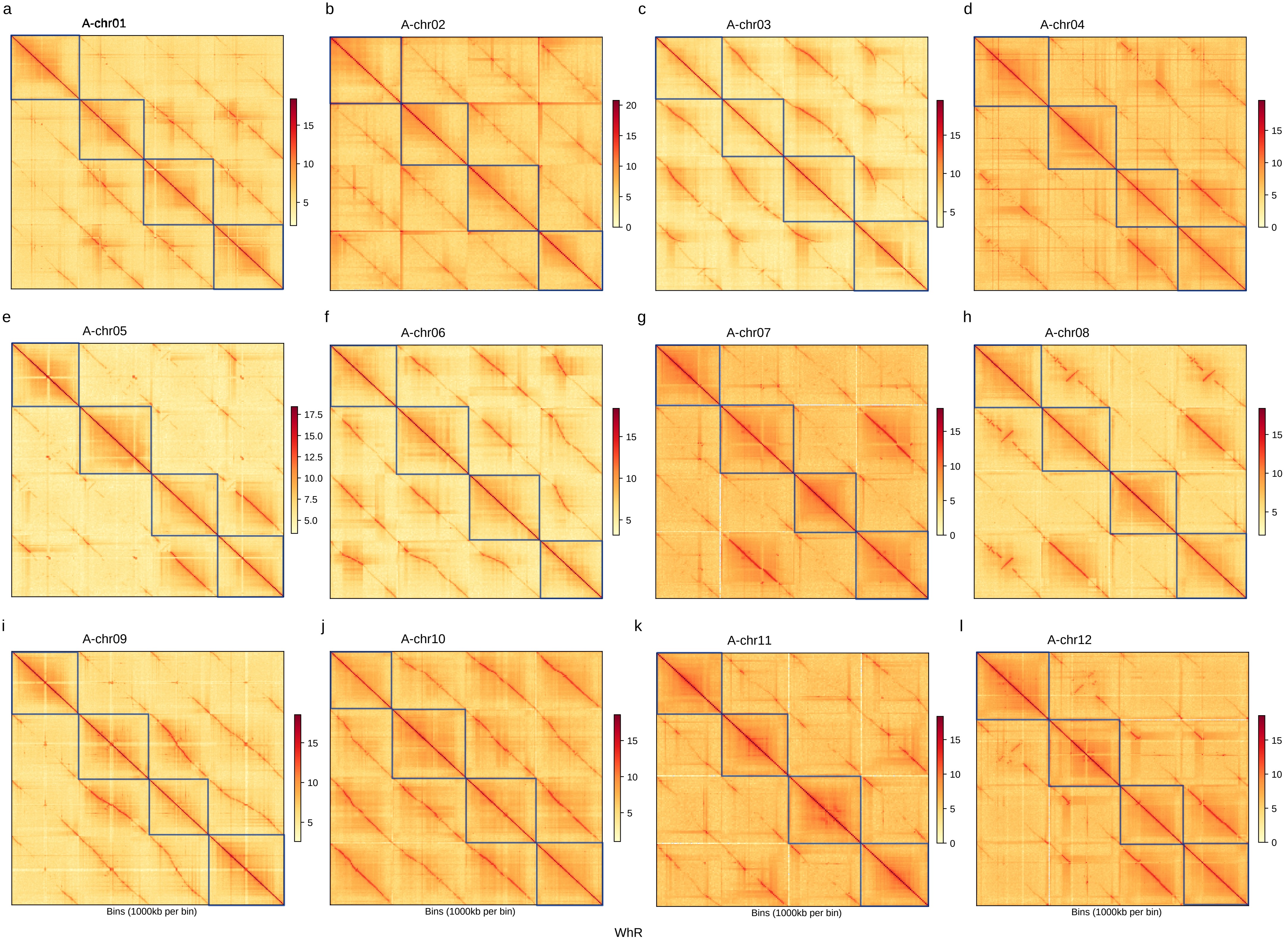

a
A-chr01
15
10
5
b
A-chr02
20
15
10
5
0
c
A-chr03
15
10
5
d
A-chr04
15
10
5
0
e
A-chr05
17.5
15.0
12.5
10.0
7.5
5.0
f
A-chr06
15
10
5
g
A-chr07
15
10
5
0
h
A-chr08
15
10
5
i
A-chr09
15
10
5
Bins (1000kb per bin)
j
A-chr10
15
10
5
Bins (1000kb per bin)
k
A-chr11
15
10
5
0
Bins (1000kb per bin)
l
A-chr12
15
10
5
0
Bins (1000kb per bin)
WhR
